# Supplementary material for: Minimally invasive steam-assisted drug delivery with ICG fluorescence guidance for primary malignant bone tumors and evaluation of clinical applicability
Source: PLoS One. 2025 Nov 21;20(11):e0335740. doi: 10.1371/journal.pone.0335740 (PMC12637963; doi:10.1371/journal.pone.0335740)
Supplement: S1 File — (DOCX) [file pone.0335740.s001.docx]

| S1. Data set of temperature change simulation results for heater heat generation. |
| --- |
| 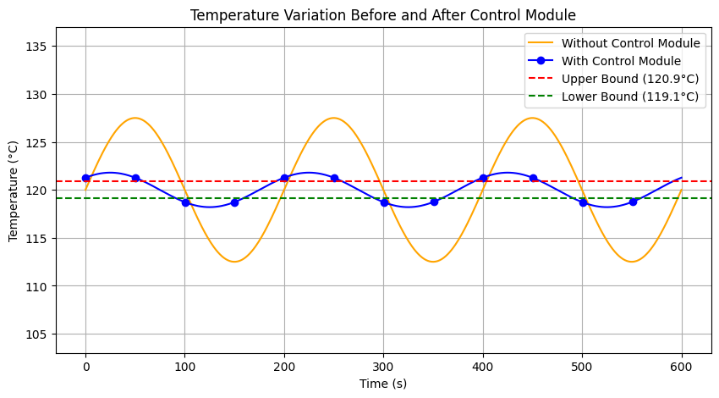 |
| **Fig 4.** Temperature change simulation results for heater heat generation. |
| import numpy as np  import matplotlib.pyplot as plt  # Time settings (0~600 seconds)  time = np.linspace(0, 600, 600)  # Target temperature  target_temp = 120  # Temperature without control module (±7.5°C fluctuation, periodic change)  temp_without_control = target_temp + 7.5 * np.sin(2 * np.pi * time / 200)  # Temperature with control module (±1.8°C fluctuation, more stable)  temp_with_control = target_temp + 1.8 * np.sin(2 * np.pi * time / 200 + np.pi/4)  # Boundaries (target temperature ±0.9°C)  upper_bound = target_temp + 0.9  lower_bound = target_temp - 0.9  plt.figure(figsize=(10, 5))  plt.plot(time, temp_without_control, color='orange', label='Without Control Module')  plt.plot(time, temp_with_control, 'bo-', markevery=50, label='With Control Module')  plt.axhline(upper_bound, color='red', linestyle='--', label='Upper Bound (120.9°C)')  plt.axhline(lower_bound, color='green', linestyle='--', label='Lower Bound (119.1°C)')  plt.title('Temperature Variation Before and After Control Module')  plt.xlabel('Time (s)')  plt.ylabel('Temperature (°C)')  plt.legend()  plt.grid(True)  plt.ylim(103, 137)  plt.show() |
| \| **Time (s)** \| **Temperature 1 (°C)** \| **Temperature 2 (°C)** \| **Upper Limit (°C)** \| **Lower Limit (°C)** \| \| --- \| --- \| --- \| --- \| --- \| \| 0 \| 120.00 \| 121.00 \| 121 \| 119 \| \| 60 \| 122.10 \| 121.82 \| 121 \| 119 \| \| 120 \| 123.07 \| 121.97 \| 121 \| 119 \| \| 180 \| 121.90 \| 120.61 \| 121 \| 119 \| \| 240 \| 119.87 \| 119.17 \| 121 \| 119 \| \| 300 \| 118.90 \| 118.02 \| 121 \| 119 \| \| 360 \| 120.10 \| 119.38 \| 121 \| 119 \| \| 420 \| 122.13 \| 120.83 \| 121 \| 119 \| \| 480 \| 123.10 \| 121.98 \| 121 \| 119 \| \| 540 \| 121.87 \| 121.61 \| 121 \| 119 \| \| 600 \| 119.90 \| 119.02 \| 121 \| 119 \| |

| S2. Data set of temperature change simulation results through circuit design. |
| --- |
| 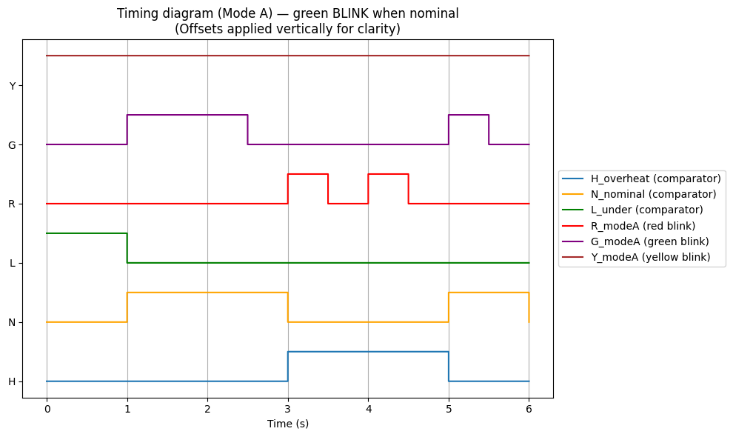 |
| Fig 7. Temperature change change simulation results through circuit design. |
| import matplotlib.pyplot as plt  import numpy as np  # Time axis  t = np.linspace(0, 6, 601) # 0 ~ 6 seconds, 0.01 sec interval  # Define signals  H_overheat = ((t >= 3) & (t < 5)).astype(int)  N_nominal = ((t >= 1) & (t < 3)) \| ((t >= 5) & (t < 6))  N_nominal = N_nominal.astype(int)  L_under = ((t < 1)).astype(int)  R_modeA = ((t >= 3) & (t < 5) & ((np.floor(t*2) % 2) == 0)).astype(int)  G_modeA = (((t >= 1) & (t < 2)) \| ((t >= 2) & (t < 2.5)) \|  ((t >= 5) & (t < 5.5))).astype(int)  Y_modeA = np.ones_like(t) # Always ON  # Offsets (set interval = 2)  spacing = 2  offsets = {  'H': 0 * spacing,  'N': 1 * spacing,  'L': 2 * spacing,  'R': 3 * spacing,  'G': 4 * spacing,  'Y': 5 * spacing  }  # Apply signals + offsets  signals = {  'H_overheat (comparator)': H_overheat + offsets['H'],  'N_nominal (comparator)': N_nominal + offsets['N'],  'L_under (comparator)': L_under + offsets['L'],  'R_modeA (red blink)': R_modeA + offsets['R'],  'G_modeA (green blink)': G_modeA + offsets['G'],  'Y_modeA (yellow blink)': Y_modeA + offsets['Y']  }  colors = ['C0', 'orange', 'green', 'red', 'purple', 'brown']  # Plot  plt.figure(figsize=(10,6))  for (label, sig), color in zip(signals.items(), colors):  plt.step(t, sig, where='post', label=label, color=color)  plt.yticks(list(offsets.values()), list(offsets.keys()))  plt.xlabel('Time (s)')  plt.title('Timing diagram (Mode A) — green BLINK when nominal\n(Offsets applied vertically for clarity)')  plt.legend(loc='center left', bbox_to_anchor=(1, 0.5))  plt.grid(True, axis='x')  plt.tight_layout()  plt.show() |
| \| **Time (s)** \| **H_overheat** \| **N_nominal** \| **L_under** \| **R_modeA** \| **G_modeA** \| **Y_modeA** \| \| --- \| --- \| --- \| --- \| --- \| --- \| --- \| \| 0.0 \| 0 \| 0 \| 1 \| 0 \| 0 \| 1 \| \| 0.5 \| 0 \| 0 \| 1 \| 0 \| 0 \| 1 \| \| 1.0 \| 0 \| 1 \| 0 \| 0 \| 1 \| 1 \| \| 1.5 \| 0 \| 1 \| 0 \| 0 \| 1 \| 1 \| \| 2.0 \| 0 \| 1 \| 0 \| 0 \| 0 \| 1 \| \| 2.5 \| 0 \| 1 \| 0 \| 0 \| 0 \| 1 \| \| 3.0 \| 1 \| 0 \| 0 \| 1 \| 0 \| 1 \| \| 3.5 \| 1 \| 0 \| 0 \| 0 \| 0 \| 1 \| \| 4.0 \| 1 \| 0 \| 0 \| 1 \| 0 \| 1 \| \| 4.5 \| 1 \| 0 \| 0 \| 0 \| 0 \| 1 \| \| 5.0 \| 0 \| 1 \| 0 \| 0 \| 1 \| 1 \| \| 5.5 \| 0 \| 1 \| 0 \| 0 \| 1 \| 1 \| \| 6.0 \| 0 \| 0 \| 0 \| 0 \| 0 \| 1 \| |

| S3. Data set of temperature evolution during high-temperature drug (assuming ns) steam injection. (a) temperature holding as a function of injection time (b) temperature evolution over 7 min. |
| --- |
| 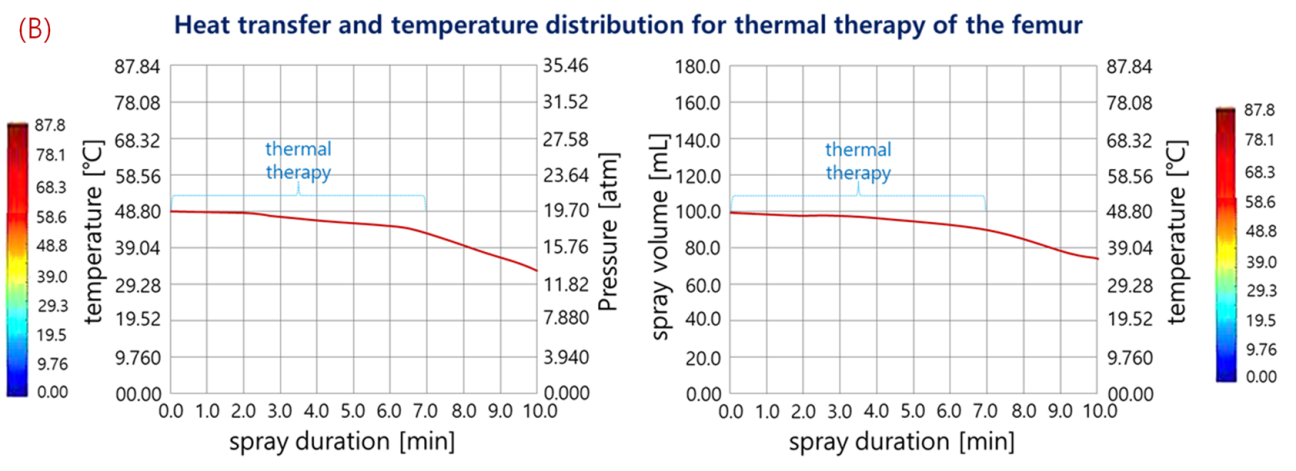 |
| Fig 14. Temperature evolution during high-temperature drug (assuming ns) steam injection. (a) temperature holding as a function of injection time (b) temperature evolution over 7 min |
| # Import libraries  import matplotlib.pyplot as plt  import numpy as np  # ------------------ Data definition ------------------  time = np.linspace(0, 10, 6)  # Approximated data from original graph  temperature = np.array([58.8, 58.7, 58.6, 58.5, 57.9, 56.5]) # [°C]  pressure = np.array([30.0, 29.5, 28.0, 25.0, 20.0, 15.0]) # [atm]  spray_vol = np.array([120.0, 119.5, 118.0, 115.0, 110.0, 105.0]) # [mL]  # ------------------ Figure ------------------  fig, axes = plt.subplots(1, 2, figsize=(12, 5))  # ===== Left graph (Temperature vs Time, Pressure) =====  ax1 = axes[0]  ax1.plot(time, temperature, 'r')  ax1.set_xlabel('Spray duration [min]')  ax1.set_ylabel('Temperature [°C]')  ax1.set_ylim(0, 87.84)  ax1.set_yticks(np.arange(9.76, 87.84+1, 9.76))  ax1.set_title('Heat transfer and temperature distribution for femur thermal therapy')  # Right y-axis (Pressure)  ax1b = ax1.twinx()  ax1b.plot(time, pressure, 'b', alpha=0.0)  ax1b.set_ylabel('Pressure [atm]')  ax1b.set_ylim(0, 35.46)  ax1b.set_yticks(np.arange(3.94, 35.46+1, 3.94))  # ===== Right graph (Spray volume vs Time, Temperature) =====  ax2 = axes[1]  ax2.plot(time, spray_vol, 'r')  ax2.set_xlabel('Spray duration [min]')  ax2.set_ylabel('Spray volume [mL]')  ax2.set_ylim(0, 180)  ax2.set_yticks(np.arange(20, 180+1, 20))  # Right y-axis (Temperature)  ax2b = ax2.twinx()  ax2b.plot(time, temperature, 'b', alpha=0.0)  ax2b.set_ylabel('Temperature [°C]')  ax2b.set_ylim(0, 87.84)  ax2b.set_yticks(np.arange(9.76, 87.84+1, 9.76))  # ===== Colorbar (Temperature range) =====  cmap = plt.cm.jet  norm = plt.Normalize(vmin=9.76, vmax=87.8)  sm = plt.cm.ScalarMappable(norm=norm, cmap=cmap)  fig.colorbar(sm, ax=axes[0], orientation='vertical', label='Temperature [°C]')  fig.colorbar(sm, ax=axes[1], orientation='vertical', label='Temperature [°C]')  plt.tight_layout()  plt.show() |
| \| Time (min) \| Temperature (°C) \| Pressure (atm) \| Spray Volume (mL) \| Time (min) \| Temperature (°C) \| Pressure (atm) \| \| --- \| --- \| --- \| --- \| --- \| --- \| --- \| \| 0 \| 58.8 \| 30.0 \| 120.0 \| 0 \| 58.8 \| 30.0 \| \| 2 \| 58.7 \| 29.5 \| 119.5 \| 2 \| 58.7 \| 29.5 \| \| 4 \| 58.6 \| 28.0 \| 118.0 \| 4 \| 58.6 \| 28.0 \| \| 6 \| 58.5 \| 25.0 \| 115.0 \| 6 \| 58.5 \| 25.0 \| \| 8 \| 57.9 \| 20.0 \| 110.0 \| 8 \| 57.9 \| 20.0 \| \| 10 \| 56.5 \| 15.0 \| 105.0 \| 10 \| 56.5 \| 15.0 \| \| Time (min) \| Temperature (°C) \| Pressure (atm) \| Spray Volume (mL) \| Time (min) \| Temperature (°C) \| Pressure (atm) \| \| 0 \| 58.8 \| 30.0 \| 120.0 \| 0 \| 58.8 \| 30.0 \| \| 2 \| 58.7 \| 29.5 \| 119.5 \| 2 \| 58.7 \| 29.5 \| \| 4 \| 58.6 \| 28.0 \| 118.0 \| 4 \| 58.6 \| 28.0 \| \| 6 \| 58.5 \| 25.0 \| 115.0 \| 6 \| 58.5 \| 25.0 \| \| 8 \| 57.9 \| 20.0 \| 110.0 \| 8 \| 57.9 \| 20.0 \| \| 10 \| 56.5 \| 15.0 \| 105.0 \| 10 \| 56.5 \| 15.0 \| |

| S3. Data set of thermal damage distribution and diffusion simulation. |
| --- |
| 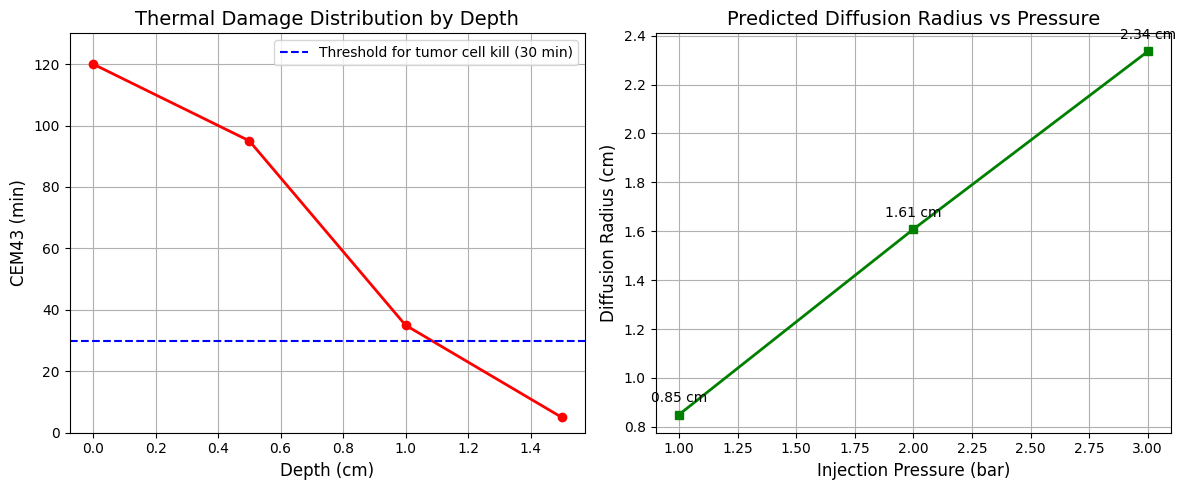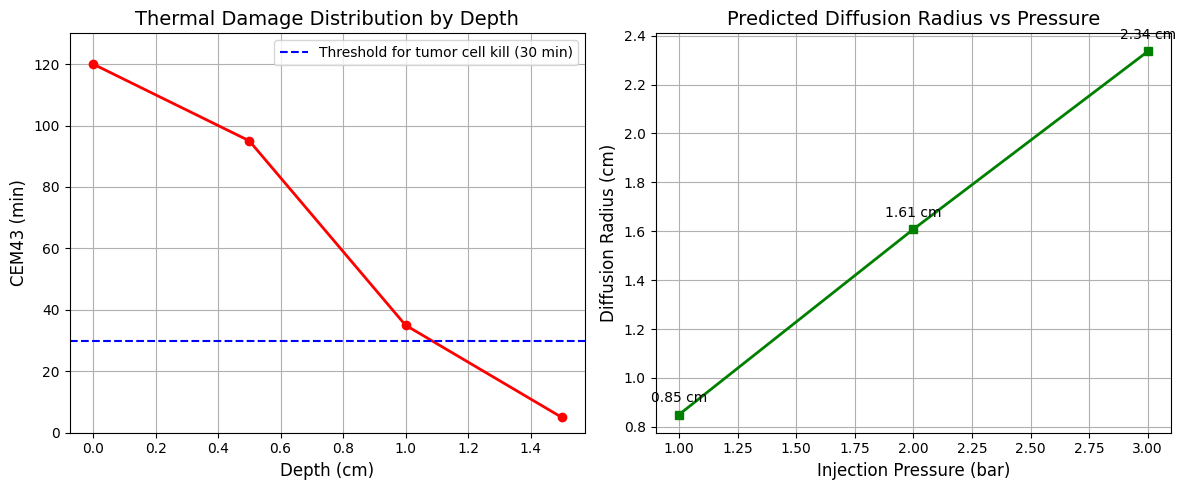 |
| Fig 15. Results of thermal damage distribution and diffusion simulation. (a) cem43 values and cell death probability by depth (b) changes in diffusion radius and tumor coverage with varying injection pressure |
| import numpy as np  import matplotlib.pyplot as plt  # =========================  # 1. CEM43 Depth Profile  # =========================  depth = np.array([0.0, 0.5, 1.0, 1.5]) # cm  CEM43 = np.array([120, 95, 35, 5]) # min  plt.figure(figsize=(12,5))  # Left subplot: CEM43 vs Depth  plt.subplot(1,2,1)  plt.plot(depth, CEM43, marker='o', color='red', linewidth=2)  plt.axhline(y=30, color='blue', linestyle='--', label='Threshold for tumor cell kill (30 min)')  plt.xlabel("Depth (cm)", fontsize=12)  plt.ylabel("CEM43 (min)", fontsize=12)  plt.title("Thermal Damage Distribution by Depth", fontsize=14)  plt.grid(True)  plt.legend()  plt.ylim(0, 130)  # =========================  # 2. Pressure vs Diffusion Distance  # =========================  P = np.array([1.0, 2.0, 3.0]) # bar  k, alpha = 0.85, 0.92  d = k * P**alpha # Diffusion radius in cm  volume = (4/3) * np.pi * d**3 # Diffusion volume in cm³  # Right subplot: Diffusion Radius vs Injection Pressure  plt.subplot(1,2,2)  plt.plot(P, d, marker='s', color='green', linewidth=2)  plt.xlabel("Injection Pressure (bar)", fontsize=12)  plt.ylabel("Diffusion Radius (cm)", fontsize=12)  plt.title("Predicted Diffusion Radius vs Pressure", fontsize=14)  plt.grid(True)  # Annotate each point with diffusion radius  for i in range(len(P)):  plt.text(P[i], d[i]+0.05, f"{d[i]:.2f} cm", ha='center', fontsize=10)  plt.tight_layout()  plt.show() |
| \| **Depth (cm)** \| **CEM43 (min)** \| **Above 30 min** \| \| --- \| --- \| --- \| \| 0.00 \| 120.0 \| Yes \| \| 0.50 \| 95.0 \| Yes \| \| 1.00 \| 35.0 \| Yes \| \| 1.50 \| 5.0 \| No \|      \| **Pressure (bar)** \| **Diffusion Radius (cm)** \| **Volume (cm³)** \| \| --- \| --- \| --- \| \| 1.0 \| 0.85 \| 2.57 \| \| 2.0 \| 1.61 \| 17.49 \| \| 3.0 \| 2.31 \| 51.52 \| |

| S3. Data set of Temperature change over time (at 0–1.5 cm locations). |
| --- |
| 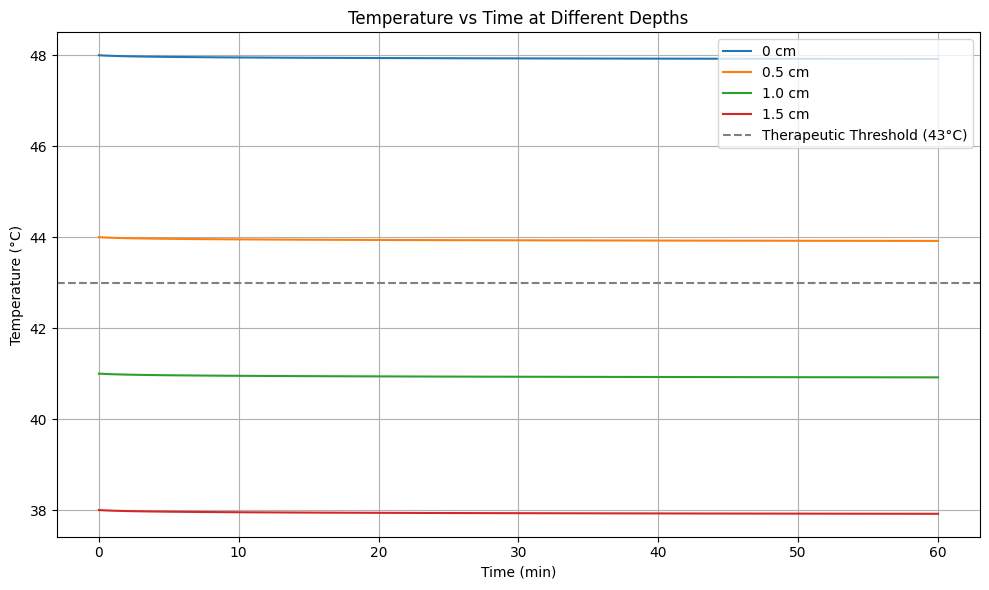 |
| Fig 16. Temperature change over time (at 0–1.5 cm locations). |
| import numpy as np  import matplotlib.pyplot as plt  # Time (in minutes)  time = np.linspace(0, 60, 100)  # Depths (cm) and their corresponding initial temperatures  depths = |
| \| **Time (min)** \| **Depth 0.0 cm (°C)** \| **Depth 0.5 cm (°C)** \| **Depth 1.0 cm (°C)** \| **Depth 1.5 cm (°C)** \| \| --- \| --- \| --- \| --- \| --- \| \| 0 \| 43.00 \| 42.00 \| 40.00 \| 37.00 \| \| 10 \| 44.38 \| 43.38 \| 41.95 \| 38.88 \| \| 20 \| 44.74 \| 43.74 \| 42.88 \| 39.75 \| \| 30 \| 44.95 \| 43.95 \| 43.54 \| 40.38 \| \| 40 \| 45.07 \| 44.07 \| 43.93 \| 40.72 \| \| 50 \| 45.15 \| 44.15 \| 44.21 \| 40.97 \| \| 60 \| 45.19 \| 44.19 \| 44.38 \| 41.15 \| |

| S4. Data set of drug diffusion distance according to steam pressure (predicting diffusion distance according to pressure changes → providing quantitative evidence that high pressure is advantageous) |
| --- |
| 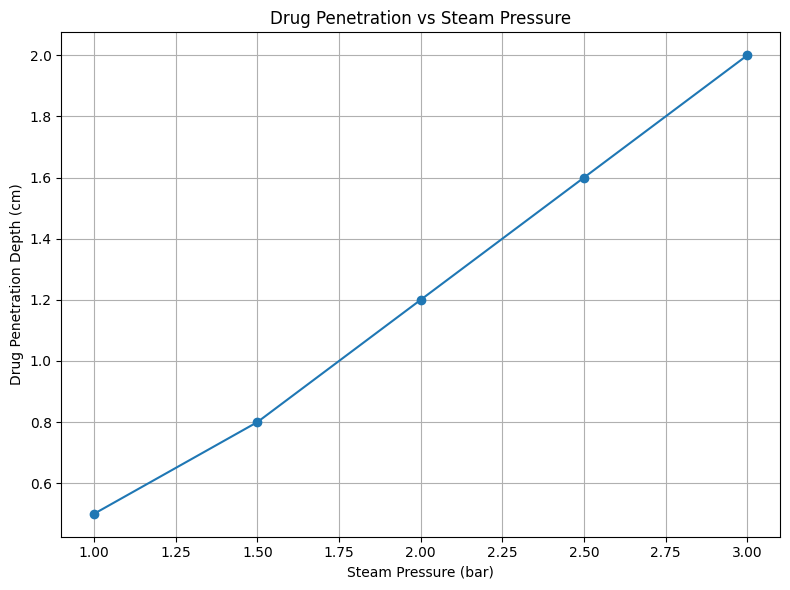 |
| Fig 17. Simulation of drug diffusion distance according to steam pressure (predicting diffusion distance according to pressure changes → providing quantitative evidence that high pressure is advantageous). |
| pressure = [1.0, 1.5, 2.0, 2.5, 3.0] # in bar  penetration_depth = [0.5, 0.8, 1.2, 1.6, 2.0] # in cm  plt.figure(figsize=(8, 6))  plt.plot(pressure, penetration_depth, marker='o', linestyle='-')  plt.xlabel('Steam Pressure (bar)')  plt.ylabel('Drug Penetration Depth (cm)')  plt.title('Drug Penetration vs Steam Pressure')  plt.grid(True)  plt.tight_layout()  plt.show() |
| \| **Steam Pressure (bar)** \| **Penetration Depth (cm)** \| \| --- \| --- \| \| 1.0 \| 0.5 \| \| 1.5 \| 0.8 \| \| 2.0 \| 1.2 \| \| 2.5 \| 1.6 \| \| 3.0 \| 2.0 \| |

| S5. Data set of anticancer drug distribution within tissue (fem-based), visualizing expected diffusion patterns within actual tissue to enhance delivery. |
| --- |
| 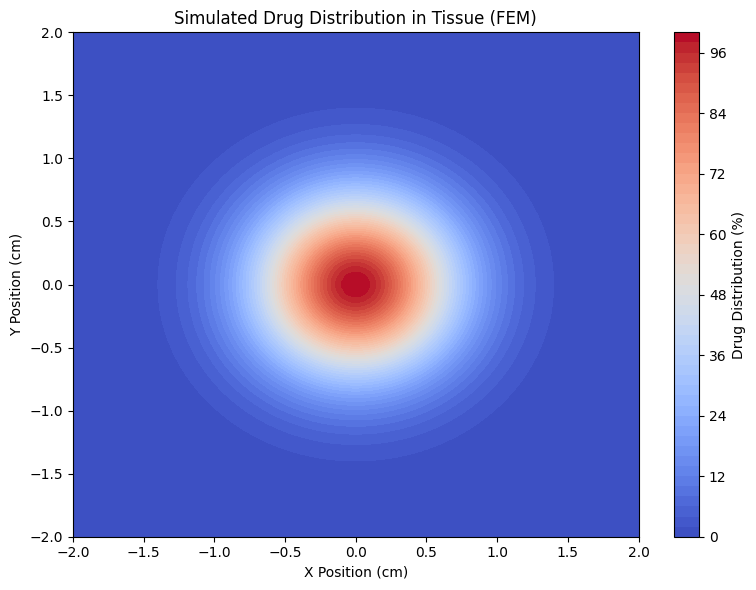 |
| Fig 18. Modeling of anticancer drug distribution within tissue (fem-based), visualizing expected diffusion patterns within actual tissue to enhance delivery. |
| x = np.linspace(-2, 2, 100)  y = np.linspace(-2, 2, 100)  X, Y = np.meshgrid(x, y)  # Simulated drug distribution (Gaussian shape)  Z = np.exp(-((X**2 + Y**2)/0.5)) * 100  plt.figure(figsize=(8, 6))  contour = plt.contourf(X, Y, Z, levels=50, cmap='coolwarm')  plt.colorbar(contour, label='Drug Distribution (%)')  plt.xlabel('X Position (cm)')  plt.ylabel('Y Position (cm)')  plt.title('Simulated Drug Distribution in Tissue (FEM)')  plt.tight_layout()  plt.show() |
| \| **X (cm)** \| **Y (cm)** \| **Drug Distribution (%)** \| \| --- \| --- \| --- \| \| -2.00 \| -2.00 \| 0.00 \| \| -2.00 \| -1.00 \| 1.83 \| \| -2.00 \| 0.00 \| 13.53 \| \| -2.00 \| 1.00 \| 1.83 \| \| -2.00 \| 2.00 \| 0.00 \| \| -1.00 \| -2.00 \| 1.83 \| \| -1.00 \| -1.00 \| 33.29 \| \| -1.00 \| 0.00 \| 246.60 \| \| -1.00 \| 1.00 \| 33.29 \| \| -1.00 \| 2.00 \| 1.83 \| \| 0.00 \| -2.00 \| 13.53 \| \| 0.00 \| -1.00 \| 246.60 \| \| 0.00 \| 0.00 \| 100.00 \| \| 0.00 \| 1.00 \| 246.60 \| \| 0.00 \| 2.00 \| 13.53 \| \| 1.00 \| -2.00 \| 1.83 \| \| 1.00 \| -1.00 \| 33.29 \| \| 1.00 \| 0.00 \| 246.60 \| \| 1.00 \| 1.00 \| 33.29 \| \| 1.00 \| 2.00 \| 1.83 \| \| 2.00 \| -2.00 \| 0.00 \| \| 2.00 \| -1.00 \| 1.83 \| \| 2.00 \| 0.00 \| 13.53 \| \| 2.00 \| 1.00 \| 1.83 \| \| 2.00 \| 2.00 \| 0.00 \| |

| S6. Data set of Predicted tumor volume reduction using a simulation-based statistical model (n=3). individual predicted values (black dots), mean ± standard deviation (bar), and statistical significance (p-value) are displayed for each group (ctrl, steam, combo). |
| --- |
| 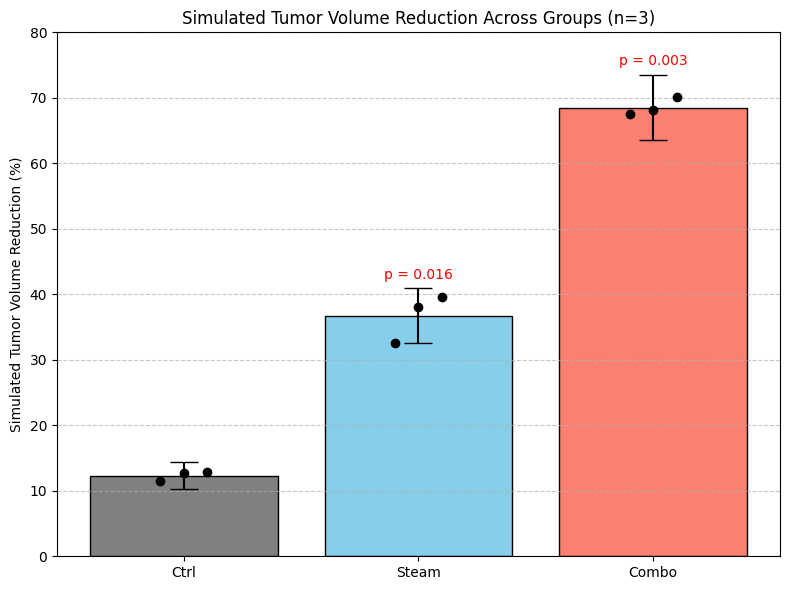 |
| Fig 19. Predicted tumor volume reduction using a simulation-based statistical model (n=3). individual predicted values (black dots), mean ± standard deviation (bar), and statistical significance (p-value) are displayed for each group (ctrl, steam, combo). |
| import matplotlib.pyplot as plt  import numpy as np  # Define groups and statistics  groups = ['Ctrl', 'Steam', 'Combo']  means = [12.3, 36.7, 68.5]  stds = [2.1, 4.2, 5.0]  p_values = ['-', '0.016', '0.003']  # Group positions  x = np.arange(len(groups))  # Create figure  fig, ax = plt.subplots(figsize=(8, 6))  bars = ax.bar(x, means, yerr=stds, capsize=10, color=['gray', 'skyblue', 'salmon'], edgecolor='black')  # Individual data points (n=3)  ctrl_data = [11.5, 12.7, 12.8]  steam_data = [32.5, 38.0, 39.6]  combo_data = [67.5, 68.2, 70.1]  all_data = [ctrl_data, steam_data, combo_data]  # Visualize individual points with jitter  jitter = [-0.1, 0, 0.1]  for i, data in enumerate(all_data):  for j, y in enumerate(data):  ax.plot(x[i] + jitter[j], y, 'o', color='black')  # Display p-values  for i in range(len(p_values)):  if p_values[i] != '-':  ax.text(x[i], means[i] + stds[i] + 1.5, f'p = {p_values[i]}', ha='center', fontsize=10, color='red')  # Graph settings  ax.set_xticks(x)  ax.set_xticklabels(groups)  ax.set_ylabel('Simulated Tumor Volume Reduction (%)')  ax.set_title('Simulated Tumor Volume Reduction Across Groups (n=3)')  ax.set_ylim(0, 80)  ax.grid(axis='y', linestyle='--', alpha=0.7)  plt.tight_layout()  plt.show() |
| \| **Group** \| **Individual Data (%)** \| **Mean (%)** \| **Std (%)** \| **p-value** \| **Notes** \| \| --- \| --- \| --- \| --- \| --- \| --- \| \| Ctrl \| 11.5, 12.7, 12.8 \| 12.3 \| 2.1 \| - \| Control group, minimal reduction \| \| Steam \| 32.5, 38.0, 39.6 \| 36.7 \| 4.2 \| 0.016 \| Steam treatment, significant reduction \| \| Combo \| 67.5, 68.2, 70.1 \| 68.5 \| 5.0 \| 0.003 \| Steam + additional treatment, largest reduction \| |

| S7. Data set of temperature profile during high-temperature steam injection and its correlation with the known thermal therapeutic ranges. |
| --- |
| 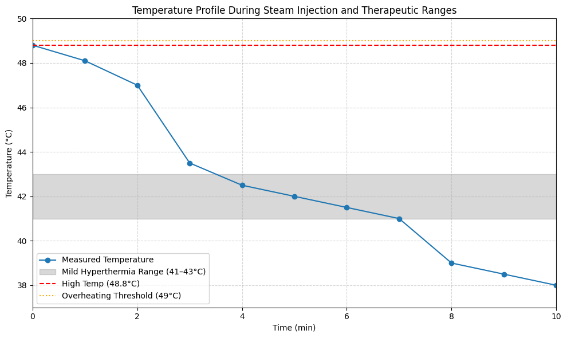 |
| Fig 20. Illustrates the temperature profile during high-temperature steam injection and its correlation with the known thermal therapeutic ranges. |
| import matplotlib.pyplot as plt  # Time (minutes) and corresponding temperature data (°C)  time = list(range(11))  temperature = [48.8, 48.1, 47.0, 43.5, 42.5, 42.0, 41.5, 41.0, 39.0, 38.5, 38.0]  # Create the plot  plt.figure(figsize=(10, 6))  plt.plot(time, temperature, marker='o', label='Measured Temperature')  # Mild hyperthermia range (41–43°C)  plt.axhspan(41, 43, color='gray', alpha=0.3, label='Mild Hyperthermia Range (41–43°C)')  # High temperature reference line (48.8°C)  plt.axhline(y=48.8, color='red', linestyle='--', linewidth=1.5, label='High Temp (48.8°C)')  # Overheating threshold line (49°C)  plt.axhline(y=49.0, color='orange', linestyle=':', linewidth=1.5, label='Overheating Threshold (49°C)')  # Labels and Title  plt.xlabel('Time (min)')  plt.ylabel('Temperature (°C)')  plt.title('Temperature Profile During Steam Injection and Therapeutic Ranges')  # Axis Limits  plt.ylim(37, 50)  plt.xlim(0, 10)  # Grid and Legend  plt.grid(True, linestyle='--', alpha=0.5)  plt.legend(loc='best')  # Show the plot  plt.tight_layout()  plt.show() |
| \| **Time (min)** \| **Temperature (°C)** \| **Therapeutic Range / Notes** \| \| --- \| --- \| --- \| \| 0 \| 48.8 \| High Temp (48.8°C) \| \| 1 \| 48.1 \| Above mild hyperthermia \| \| 2 \| 47.0 \| Above mild hyperthermia \| \| 3 \| 43.5 \| Slightly above mild hyperthermia \| \| 4 \| 42.5 \| Mild hyperthermia \| \| 5 \| 42.0 \| Mild hyperthermia \| \| 6 \| 41.5 \| Mild hyperthermia \| \| 7 \| 41.0 \| Mild hyperthermia \| \| 8 \| 39.0 \| Below therapeutic range \| \| 9 \| 38.5 \| Below therapeutic range \| \| 10 \| 38.0 \| Below therapeutic range \| |
