# Supplementary material for: Minimally invasive steam-assisted drug delivery with ICG fluorescence guidance for primary malignant bone tumors and evaluation of clinical applicability
Source: PLoS One. 2025 Nov 21;20(11):e0335740. doi: 10.1371/journal.pone.0335740 (PMC12637963; doi:10.1371/journal.pone.0335740)
Supplement: Attachment — Submitted filename: REVIEWERS REPORT.docx [file pone.0335740.s002.docx]

I acknowledge the efforts of the authors in their attempt for the development and preclinical evaluation of a novel drug delivery system for treating metastatic bone tumors using high-temperature steam to deliver anticancer drugs. The concept is interesting and potentially valuable. The authors aim to address the limitations of conventional cementoplasty by enhancing drug diffusion and incorporating thermal ablation. However, the manuscript has several significant shortcomings in experimental design, data presentation, and overall clarity. It reads more like a preliminary report than a robust scientific study.

**Major Concerns**

1. **Rationale and Background:**
   - The introduction states that malignant bone tumors are rare tumors with a poor prognosis, with a reported 5-year survival rate of 65.3% and 10-year survival rate of 58.0% for malignant bone tumors in the United States in 2024 [1]. The introduction also mentions metastatic bone tumors, the most common form of these bone tumors, cause severe pain, gait disturbance, and pathologic fractures. The authors need to clarify whether the study focuses on primary or metastatic bone tumors and provide more context on the specific clinical need being addressed. It is essential to clarify if metastatic bone tumors are indeed the most common type, as primary bone cancers (like osteosarcoma) are much rarer.
   - The rationale for using cementoplasty as a comparator is not clearly justified. The manuscript needs to explain why cementoplasty is the primary conventional treatment being targeted for replacement, especially considering the hardening and fracture prevention aspects it offers.
   - The introduction includes a reference to United States survival rates for a clinical purpose when the study and authors are based in Korea. This disconnect requires justification.
2. **System Design and Drug Delivery:**
   - The description of the STEAM system is incomplete and confusing. The design looks incomplete. The main player is the drug, and is largely missing! Is this system a simple mechanical tool, or a classical system incorporating drug delivery manipulation?
   - Figure 3, illustrating the system design, lacks detail. The role of the anticancer drug within the system is not clearly defined. Is the system a simple mechanical tool, or does it incorporate drug delivery manipulation? This needs clarification. The authors lose focus on the design of their system, which is what the scientific community would like to understand and how this fits into a useful system in replacement of the conventional - this focus is completely lost!
   - There are concerns about the effect of high temperature (120°C) on the efficacy and degradation of the therapeutic agents. This critical point is not adequately addressed. The study mentions "The anticancer drug filled in the water tank is heated to 120 through the steam generator". There are concerns about this temperature and its potential degradation of the therapeutic agents.
3. **Experimental Methods:**
   - The animal model (mini-pig femur) and experimental setup are described, but crucial details are missing. There's a need to clarify the method used to induce or simulate bone tumors in the mini-pig model, or if healthy bone was used.
   - The study mentions "This study was conducted using animal experiments, and all procedures were carried out in strict compliance with relevant animal welfare laws", the paper does not follow a standard scientific reporting - no ethical reporting is mentioned throughout the manuscript raising a question about transparency. There is an urgent need for more detailed reporting on animal care and ethical approval.
   - The description of the preclinical test lacks essential details regarding the number of animals used, the specific drug and dosage used, and the method of tumor implantation (if applicable).
4. **Results and Data Presentation:**
   - The results focus primarily on temperature changes during steam injection. While this is important, the manuscript lacks data on the efficacy of the drug delivery system in killing tumor cells or reducing tumor growth.
   - A big expectation was missed! It's crucial to include the outcomes/findings of the experiment - the efficacy and potential downsides (limitation) of the investigation. The study is simply all about "temperature evolution during high-temperature anticancer drug steam injection".
   - There needs to be a comparative analyses to test efficacy of this method against the conventional method. The authors claimed many downsides of the existing methods and resulted to introducing an expectedly better and novel approach. To ensure rigor of this technique and acceptability of the acclaimed applicability, it is important that the authors show some comparisons with the existing or conventional methods and how this novel method is better.
   - The use of an IR camera to analyze injection status is mentioned, but the images are not clearly presented or interpreted.
   - Table 2 presents data on temperature, pressure, and spray volume over time, but it is difficult to assess the clinical significance of these measurements without corresponding data on tumor response.
5. **Discussion and Conclusion:**
   - The discussion acknowledges the need for further clinical trials but does not adequately address the limitations of the current study. In fact, where are the efficacy results?? The authors need to address whether the animal experiments follow standard scientific reporting. The whole writeup was a typical a news-reported style!
   - The conclusion overstates the potential clinical value of the system without sufficient evidence from the preclinical experiments.
6. **Writing and Clarity:**
   - The manuscript suffers from poor organization and a lack of clarity. The writing style is more akin to a news report than a scientific paper, as earlier mentioned.
   - There are instances where the authors lose focus on the design of their system. The importance of Figure 2 is not clear.

**Conclusion**

While the concept presented in this manuscript has merit, the current study is not sufficiently rigorous to support strong conclusions about the potential of the STEAM-based drug delivery system. Significant revisions are needed, including more experiments, to address the identified concerns before this manuscript can be considered for publication.
