# Supplementary material for: Minimally invasive steam-assisted drug delivery with ICG fluorescence guidance for primary malignant bone tumors and evaluation of clinical applicability
Source: PLoS One. 2025 Nov 21;20(11):e0335740. doi: 10.1371/journal.pone.0335740 (PMC12637963; doi:10.1371/journal.pone.0335740)
Supplement: Attachment — Submitted filename: commnts #2.docx [file pone.0335740.s004.docx]

**Reviewer comments #1**

**Comments 1 :**

I thank the editors for the opportunity to review this interesting and innovative study. I commend the authors for their rigorous work and propose major revisions related to the manuscript's writing, organization, and data availability. This manuscript presents a novel and creative approach to the treatment of bone cancer, with a promising steam-based drug delivery system evaluated in a porcine preclinical model. The use of pigs enhances the translational potential of the study, and the authors demonstrate the ability to control and maintain temperatures relevant to anticancer hyperthermia. The thermal modeling predictions are also validated, which is a strength of the study. However, the drug delivery aspect of the STEAM system would benefit from additional validation. Specifically:

**Answer 1 :**

Thank you very much for your careful review of my manuscript. I will do my best to revise the manuscript and provide a response based on the points you have raised.

**Comments 2 :**

Is the antibody still active following steam exposure?

Answer 1:

This study focused on evaluating the technical performance of the high-temperature steam injection system, and antibody-based drugs were not used. Antibodies are generally known to undergo structural changes and lose activity under high-temperature conditions. Therefore, future research will aim to develop heat-resistant antibody formulations capable of maintaining stability under steam conditions, along with designing appropriate drug delivery systems. Follow-up experiments using tumor models are planned to verify antibody stability and therapeutic efficacy in high-temperature environments. Please refer to lines 516–558, Table 14, Figure 20, and Table 15 in the discussion section.

**Comments 2 :**

Is the drug bioavailable to the bone tumor microenvironment using this system? If not, does steam alone offer sufficient therapeutic benefit for treating bone cancer?

**Answers 2 :**

This study did not directly analyze drug bioavailability within the tumor microenvironment; rather, it focused on evaluating the fundamental performance and temperature distribution of the high-temperature steam injection system in healthy bone tissue. The experimental results showed that the targeted lesion area reached a maximum temperature of 48.8 °C, and the therapeutic hyperthermia range of 41–43 °C was maintained for approximately 37 minutes, meeting the conditions necessary to induce tumor cell necrosis. No overheating above 49 °C was observed, indicating a low risk of damage to normal tissue. Therefore, the steam itself may exert a therapeutic effect through hyperthermia, potentially contributing to tumor suppression. Future studies will employ tumor-bearing animal models to comprehensively analyze how steam-based drug delivery affects intratumoral drug distribution, tissue penetration, and tumor cell death. Additionally, combination therapy strategies using antibodies or immunotherapeutics are also under consideration. Please refer to lines 514–558, Table 14, Figure 20, and Table 15 in the discussion section.

**Comments 3:**

Addressing these questions through further experimentation would significantly enhance the impact and translational relevance of this work.

**Answer 3:**

Please refer to lines 514–558, Table 14, Figure 20, and Table 15 in the discussion section. We fully agree with your comments. This study represents an initial stage focused on demonstrating technical feasibility and obtaining fundamental data. Future preclinical experiments using tumor-bearing animal models are planned to comprehensively evaluate the effects of steam-based drug injection on the tumor microenvironment, including thermal impact, drug diffusion range, antibody stability, and tumor cell death induction. Additionally, by analyzing biological response indicators such as heat shock protein (HSP) expression, tumor necrosis extent, and immune cell infiltration, we aim to further clarify the potential clinical applicability of this approach.

**Comments 4 :**

The manuscript would also benefit from substantial revisions to its writing and organization. I was particularly impressed by the agreement between the STEAM system's performance and the thermal modeling. To improve clarity, I recommend presenting the predicted and achieved temperatures within the same figure. In addition, Figures 1 and 2 could be combined and condensed for better flow and impact.

**Answer 4 :**

We appreciate the reviewer’s suggestion to present both predicted and measured temperatures in a single figure, which would indeed improve clarity. However, the thermal modeling and data acquisition software used in this study do not support direct overlay of predicted results with experimental measurements. Artificially combining them could compromise the integrity of the original data. We plan to adopt compatible analysis software in future work to incorporate the reviewer’s recommendation. As a practical adjustment for the current manuscript, Figures 1 and 2 have been merged into a single figure, now presented as Figure 1:

Figure 1. Conventional bone tumor treatment and immune evasion mechanism via PD-1/PD-L1 pathway.

(a) Conventional treatment of bone tumors using drilling and injecting anticancer drugs.

(b) Mechanism of immune evasion via PD-1/PD-L1 interaction.

**Comments 5 :**

The discussion section should be expanded considerably. I suggest including a concise summary of the study’s key findings and more thoroughly contextualizing the significance of the work within the broader field of bone cancer treatment and drug delivery. While the authors address some of these points, a more comprehensive overview of the current landscape would help underscore the novelty and potential of this approach. The manuscript would also benefit from close proofreading, ideally with the assistance of a colleague or a language tool (e.g., Grammarly), to improve clarity and consistency.

**Answer 5 :**

We sincerely appreciate the reviewer’s insightful suggestion to emphasize the study’s key findings within the broader context of bone cancer treatment and drug delivery. In the revised manuscript, we have addressed this point by clearly highlighting current research trends as well as the novelty and potential impact of our work. Additionally, to enhance clarity and consistency, we thoroughly reviewed the manuscript using peer feedback and language editing tools. We remain committed to further improving the manuscript’s quality up to the point of publication.

**Comments 6 :**

Finally, I strongly recommend including a supplementary table and making the software and raw data publicly available, for example via a GitHub repository. The number of pigs used should be stated explicitly, and the statistical reporting should be more detailed, ideally including error bars, p-values, and individual data points where appropriate.

**Answer 6 :**

We sincerely appreciate your valuable suggestions. We have added a table (Table 13) comparing our measured temperatures with the hyperthermia ranges reported in the literature, along with figures illustrating the temperature variations predicted by simulations. Please refer to the discussion section, lines 475–507, equations (12) and (13), Figures 17 and 19, and Tables 12 and 13.

This study was conducted as a preliminary proof-of-concept experiment using a single mini-pig, which has been clearly stated in the manuscript. However, we performed mathematical analyses assuming three animals to estimate statistical outcomes and predicted results for a sample size of three. Please see the discussion section, lines 432–433 and 475–505, equations (12) and (13), Figures 17 and 19, and Tables 12 and 13. These results demonstrate an approach that minimizes animal sacrifice while still allowing estimation of outcomes through statistical modeling.

No custom software was developed for this study; only commercially available analytical tools were used, and no code repository (e.g., GitHub) was employed. Raw experimental data and analysis results are available upon request. In future work, we plan to increase the sample size and include p-values, error bars, and individual data points to provide more quantitative and reliable results.

**Reviewer comments #2**

**Comments 0 :**

I acknowledge the efforts of the authors in their attempt for the development and preclinical evaluation of a novel drug delivery system for treating metastatic bone tumors using high-temperature steam to deliver anticancer drugs. The concept is interesting and potentially valuable. The authors aim to address the limitations of conventional cementoplasty by enhancing drug diffusion and incorporating thermal ablation. However, the manuscript has several significant shortcomings in experimental design, data presentation, and overall clarity. It reads more like a preliminary report than a robust scientific study.

**Answer 0 :**

Thank you very much for your careful review of our manuscript. We will do our utmost to revise the manuscript and provide a response based on your valuable comments.

**【Major Concerns】**

**Comments 1 : Rationale and Background:**

• The introduction states that malignant bone tumors are rare tumors with a poor prognosis, with a reported 5-year survival rate of 65.3% and 10-year survival rate of 58.0% for malignant bone tumors in the United States in 2024 [1]. The introduction also mentions metastatic bone tumors, the most common form of these bone tumors, cause severe pain, gait disturbance, and pathologic fractures. The authors need to clarify whether the study focuses on primary or metastatic bone tumors and provide more context on the specific clinical need being addressed. It is essential to clarify if metastatic bone tumors are indeed the most common type, as primary bone cancers (like osteosarcoma) are much rarer.

• The rationale for using cementoplasty as a comparator is not clearly justified. The manuscript needs to explain why cementoplasty is the primary conventional treatment being targeted for replacement, especially considering the hardening and fracture prevention aspects it offers.

• The introduction includes a reference to United States survival rates for a clinical purpose when the study and authors are based in Korea. This disconnect requires justification.

**Answer 1 :**

We apologize for any confusion caused by the reviewer’s question. To clarify the research objectives and scope, we have revised the introduction. This study focuses on the development of a high-temperature, high-pressure steam injection device and its preclinical evaluation in healthy animal bone, and it does not directly assess therapeutic effects on cancer tissue. Accordingly, we have also clarified the rationale for comparison with conventional treatments such as cementoplasty and provided a clear research background. We will continue to ensure that the study content is communicated as clearly as possible. Please refer to the revised sections in the introduction: lines 36–59 and lines 83–93.

**Comments 2 (System Design and Drug Delivery) :**

• The description of the STEAM system is incomplete and confusing. The design looks incomplete. The main player is the drug, and is largely missing! Is this system a simple mechanical tool, or a classical system incorporating drug delivery manipulation?

• Figure 3, illustrating the system design, lacks detail. The role of the anticancer drug within the system is not clearly defined. Is the system a simple mechanical tool, or does it incorporate drug delivery manipulation? This needs clarification. The authors lose focus on the design of their system, which is what the scientific community would like to understand and how this fits into a useful system in replacement of the conventional - this focus is completely lost!

**Answer 2 :**

We apologize for any confusion and sincerely appreciate your precise comments. Our study assumes that the drug is placed in a water tank and heated to 120 °C during administration. It is crucial that this temperature is maintained consistently throughout the treatment, as any increase beyond or decrease below 120 °C could interfere with the therapeutic process. Therefore, we proposed a system capable of real-time temperature monitoring and providing temperature feedback to prevent undesired fluctuations. For further details, please refer to the following sections in the revised manuscript:

• Introduction: lines 65–78.

• System manufacturing and drug delivery methods: lines 165–230, Equations (6)–(9), Figures 4–7, Tables 2–3

• Experiment environment and results: lines 274–316, Tables 5–7, Figures 9–11

• Discussion: lines 419–432.

• There are concerns about the effect of high temperature (120°C) on the efficacy and degradation of the therapeutic agents. This critical point is not adequately addressed. The study mentions "The anticancer drug filled in the water tank is heated to 120 through the steam generator". There are concerns about this temperature and its potential degradation of the therapeutic agents. In this study, actual anticancer drugs were not used, and the evaluation of drug stability at high temperatures (120 °C) was beyond the scope of this work. However, we acknowledge the reviewer’s point regarding potential temperature fluctuations and drug degradation during administration from a high-temperature water tank. This issue will be carefully addressed in future studies involving actual anticancer agents. We appreciate this valuable suggestion. For details, please refer to the revised Discussion section, lines 575–580.

**Comments 3 (Experimental Methods) :**

• The animal model (mini-pig femur) and experimental setup are described, but crucial details are missing. There's a need to clarify the method used to induce or simulate bone tumors in the mini-pig model, or if healthy bone was used.

**Answer 3 :**

We apologize for any confusion and appreciate your insightful comments. In this study, no tumors were induced; experiments were conducted using the healthy femur of a minipig. The primary objective was to evaluate the physical performance of the proposed steam injection system, and the tumor environment was not replicated in the animal model. Instead, the potential drug diffusion during anticancer agent injection was analyzed indirectly through mathematical modeling and simulations. For detailed information, please refer to the Experiment Environment and Results section, lines 317–319, lines 387–397, lines 399–417, Equations (10)–(11), Tables 9 and 10, and Figure 15. Additionally, see the Discussion section, lines 434–444, 449–452, 456–460, 465–472, Figures 16–18, and Table 11.

**Comments 4 :**

The study mentions "This study was conducted using animal experiments, and all procedures were carried out in strict compliance with relevant animal welfare laws", the paper does not follow a standard scientific reporting - no ethical reporting is mentioned throughout the manuscript raising a question about transparency. There is an urgent need for more detailed reporting on animal care and ethical approval.

**Answer 4 :**

All animal experiments were conducted with the approval of the Institutional Animal Care and Use Committee and in compliance with both domestic and international animal welfare regulations. The animals’ conditions were monitored before, during, and after the experiments. Anesthesia was administered using [anesthetic name] at the specified dosage. At the conclusion of the experiments, humane euthanasia was performed.

For detailed information, please refer to the System Manufacturing and Drug Delivery Methods section, lines 234–259, Figure 8, Table 4, and lines 263–269. Additionally, see the Experiment Environment and Results section, lines 333–336, and lines 339–340.

**Comments 5 :**

The description of the preclinical test lacks essential details regarding the number of animals used, the specific drug and dosage used, and the method of tumor implantation (if applicable).

**Answer 5 :**

This study was conducted using a single mini-pig, and no anticancer drugs were administered. Instead, a solution of saline mixed 1:1 with black ink was converted into steam and injected to mimic drug distribution characteristics. The performance of the system was evaluated by observing the distribution of the mixed ink (as a fluorescent tracer) and saline within the femur.

No tumor implantation or induction was performed, and the therapeutic effects of anticancer drugs were predicted through mathematical analysis and simulation. For detailed information, please refer to the Experiment Environment and Results section, lines 317–319, lines 387–417, equations (10)–(11), Tables 9–10, and Figure 15. Additionally, see the Discussion section, lines 434–444, Figure 16, Table 11, lines 449–452, Figure 17, lines 456–460, Figure 18, and lines 465–472.

**Comments 6 (Results and Data Presentation) :**

• The results focus primarily on temperature changes during steam injection. While this is important, the manuscript lacks data on the efficacy of the drug delivery system in killing tumor cells or reducing tumor growth.

**Answer 6 :**

We sincerely appreciate the opportunity to revise the manuscript and your insightful comments. The revised content can be found in the Experiment Environment and Results section, lines 317–319, lines 387–417, equations (10)–(11), Tables 9–10, and Figure 15.

This study was a preliminary experiment conducted on healthy bone; thus, no direct data on tumor cell death or tumor growth inhibition were obtained. However, the measured temperature profiles were applied to the thermal damage model (CEM43) and drug diffusion simulations to indirectly evaluate the potential therapeutic effect. The analysis indicated that under the experimental conditions, the calculated CEM43 values within 1 cm from the injection site exceeded the threshold for tumor cell death, and the predicted drug diffusion radius at pressures above 2 bar was sufficient to cover the target tumor size. These results suggest that the developed system has potential to achieve therapeutic effects in tumor models.

**Comments 7 :**

A big expectation was missed! It's crucial to include the outcomes/findings of the experiment - the efficacy and potential downsides (limitation) of the investigation. The study is simply all about "temperature evolution during high-temperature anticancer drug steam injection".

**Answer 7 :**

We sincerely appreciate the reviewer’s insightful comment. We agree that the previous version lacked a sufficient description of the therapeutic efficacy and limitations of our study. In response, we have revised the Results and Discussion sections to clarify that our study was designed not only to observe temperature changes, but also to assess the potential of our system to deliver anticancer agents effectively via high-temperature steam into bone tissue. We added new tables and graphs showing temperature distribution, steam diffusion patterns, and their potential biological implications. Further, we discussed the limitations of not using a tumor-bearing model and proposed future studies to validate the efficacy in metastatic bone tumor models. Thank you again for pointing out this important issue. Please refer to **lines 434–472, Figures 16–18, and Table 11** in the Discussion section.

**Comments 8** :

There needs to be a comparative analyses to test efficacy of this method against the conventional method. The authors claimed many downsides of the existing methods and resulted to introducing an expectedly better and novel approach. To ensure rigor of this technique and acceptability of the acclaimed applicability, it is important that the authors show some comparisons with the existing or conventional methods and how this novel method is better.

**Answer 8:**

This study does not include direct experimental data comparing the proposed system with conventional liquid injection methods. This is because the study focused on a preclinical evaluation of the steam injection system’s delivery performance and diffusion characteristics in healthy bone, rather than on validating the therapeutic efficacy of actual anticancer drug administration. Instead, we have detailed the limitations of conventional liquid injection methods reported in previous studies and the literature—such as restricted drug diffusion rate and limited distribution—and supplemented this with mathematical modeling and simulations to demonstrate that the gas-phase drug in our steam-based delivery system can diffuse more rapidly and broadly within tissue.

Moreover, the Results and Discussion sections reflect how the steam injection system could theoretically improve drug delivery efficiency and tissue penetration depth compared to conventional methods, based on indirect analyses. We also clearly stated that future studies will conduct direct comparative experiments using animal tumor models to objectively evaluate the efficacy of this system relative to conventional treatments, establishing a foundation for subsequent research.

Thus, this paper emphasizes the design and performance evaluation of a novel steam-based drug delivery system, while clarifying to readers that comparisons with conventional methods and clinical applicability remain important objectives for future studies. For the supplementary details, please refer to the Experiment Environment and Results section: lines 317–319, lines 387–417, equations (10)–(11), Tables 9–10, and Figure 15. We have supplemented the manuscript by presenting comparative analyses with conventional systems to highlight the advantages of our proposed system. Please refer to the **Discussion section: lines 559–570, and Table 16** for the revised content.

**Comments 9 :**

The use of an IR camera to analyze injection status is mentioned, but the images are not clearly presented or interpreted.

**Answer 9 :**

The original purpose of using the IR camera was to evaluate the injection performance of ink inside the bone by employing a fluorescent contrast agent and monitoring it via the IR camera. This detail appears to have been omitted. Please refer to the **Experiment environment and results session: lines 353–358** for the relevant information.

**Comments 10 :**

Table 2 presents data on temperature, pressure, and spray volume over time, but it is difficult to assess the clinical significance of these measurements without corresponding data on tumor response.

**Answer 10 :**

The data in **Table 8 (formerly Table 2)** on temperature, pressure, and spray volume over time were intended to evaluate the physical performance of the system. Tumor response data were not directly measured in this study; however, the clinical potential was indirectly assessed using these physical performance metrics combined with simulation results based on established drug diffusion theories. For detailed information, please refer to the **Experiment environment and results session: lines 317–319, lines 387–417, Equations (10)–(11), Tables 9–10, Figure 15**. Additionally, see the **Discussion session: lines 434–444, Figure 16, Table 11, lines 449–452, Figure 17, lines 456–460, Figure 18, and lines 465–472**.

**Comments 11 (Discussion and Conclusion):**

• The discussion acknowledges the need for further clinical trials but does not adequately address the limitations of the current study. In fact, where are the efficacy results?? The authors need to address whether the animal experiments follow standard scientific reporting. The whole writeup was a typical a news-reported style!

**Answer 11 :**

This study is a preliminary proof-of-concept experiment conducted on healthy bone. Direct in vivo results on anticancer efficacy are not included, and efficacy evaluation will be conducted in future preclinical and clinical studies using tumor models. However, in this study, mathematical modeling and simulations were used to indirectly demonstrate potential drug diffusion and anticancer effects. All procedures adhered to standard scientific reporting guidelines and ethical regulations. For detailed information, please refer to the **Experiment environment and results session: lines 317–319, lines 387–417, Equations (10)–(11), Tables 9–10, Figure 15**. Additionally, see the **Discussion session: lines 434–444, Figure 16, Table 11, lines 449–452, Figure 17, lines 456–460, Figure 18, and lines 465–472**.

**Comments 12 :**

The conclusion overstates the potential clinical value of the system without sufficient evidence from the preclinical experiments.

**Answer 12 :**

We appreciate the reviewer’s comments. In this study, predictive results were obtained through mathematical modeling, and animal experiments were also conducted to acquire actual data (see lines 234–259, Figure 8, Table 4). In the conclusion, we clarified that this study represents a proof-of-concept stage and that the current results provide preliminary evidence regarding the system’s physical performance and potential drug diffusion. The actual clinical applicability must be verified through future tumor model experiments and clinical trials. To prevent potential confusion, we added this clarification in the

Conclusion session: lines 593–596.

**Comments 13 (Writing and Clarity):**

• The manuscript suffers from poor organization and a lack of clarity. The writing style is more akin to a news report than a scientific paper, as earlier mentioned.

**Answer 13 :**

The overall structure of the manuscript has been reorganized to follow the standard scientific format (Introduction–Methods–Results–Discussion–Conclusion). Exaggerated expressions and subjective tones have been removed. All descriptions have been revised to ensure objective, data-driven narration, and explanations of visual materials (tables and figures) have been made more detailed and precise.

**Comments 14 :**

There are instances where the authors lose focus on the design of their system. The importance of Figure 2 is not clear.

**Answer 14 :**

We recognized that certain parts of the manuscript somewhat diluted the focus on system design, particularly regarding Figure 2 (renumbered as Figures 1 and 2), whose role and significance might not have been clearly conveyed, potentially causing confusion for readers.

Accordingly, the following revisions were made:

1. Enhanced description of system design: The text related to Figure 2 (renumbered as Figures 1 and 2) was supplemented to clearly indicate that the figure visually illustrates the system’s key components and operating principles (see Figures 5, 6, and 10).
2. Emphasis on the importance of Figure 2: The figure is highlighted as a critical reference that allows readers to understand the overall structure and workflow of the steam injection system developed in this study.
3. Strengthened linkage between the figure and text: References to Figure 2 in the manuscript were clarified and specified so that readers can immediately grasp the main functions and design intent of the system through the figure.
4. Overall improvement: These revisions help readers better understand the system design and increase the manuscript’s overall clarity and focus.

**Comments 15 (Conclusion) :**

While the concept presented in this manuscript has merit, the current study is not sufficiently rigorous to support strong conclusions about the potential of the STEAM-based drug delivery system. Significant revisions are needed, including more experiments, to address the identified concerns before this manuscript can be considered for publication.

**Answer 14 :**

This study represents an early-stage, preliminary proof-of-concept investigation of a steam-based drug delivery system. The current research focused primarily on evaluating the system’s physical performance and conducting mathematical simulations, which imposes limitations on drawing strong conclusions regarding clinical applicability. Accordingly, the manuscript clarifies the following points:

1. The study focused on assessing the physical performance using healthy mini-pig bones, and it clearly states that actual tumor models or evaluations of anticancer efficacy were not included.
2. Direct in vivo evidence of anticancer effects is planned for future studies; at present, the potential therapeutic outcomes were assessed indirectly through mathematical modeling and simulations.
3. The study’s limitations are explicitly acknowledged, and the discussion highlights that additional experiments and clinical trials are essential in follow-up research.
4. When appropriate, future experimental and research plans are outlined.

Thus, this manuscript serves as a foundational step in steam-based drug delivery system research, emphasizing that rigorous subsequent experiments and clinical validation are necessary to confirm its utility.

**Reviewer comments #3**

**Comments 0 :**

The authors of the article "Minimally invasive drug delivery system using hightemperature and high-pressure steam for treating transmetastatic bone tumors and evaluation of clinical applicability" have proposed a new innovative method, The STEAM-based anticancer drug injection system, for delivering drugs to the bone. The data shows its effectiveness in delivering drugs than the conventional methods. However the quality of the article can be improved.

**Answer 0 :**

Thank you very much for taking the time to review my manuscript with such care. I will carefully consider your comments and do my utmost to revise the manuscript and provide thorough responses accordingly.

**comments 1 :**

the authors have to mention the number of animals used for the preclinical study and draw statistical conclusions for the data.

**Answer 1 :**

Reviewer 1 raised a similar question, so we added a sentence in the discussion session (lines 432–433). We also added a table comparing the measured temperature results with the reported hyperthermia treatment ranges (Table 7) and provided a figure illustrating the temperature changes based on simulation (Figure 19). Please refer to the discussion session, lines 473–505, Table 12, Table 13, and Equations (12–13) for details. This study was conducted as a preliminary proof-of-concept experiment using a single mini-pig, and this has been explicitly stated in the main text.

**Comments 2 :**

"When PD-L1 on cancer cells binds to Programmed Death-1 (PD-1) on T cells, the cancer cells are killed by the diminished function of the T cells, as shown in Figure 2" I think it is "Cancer cells are not killed" in the place of "cancer cells are killed"

**Answer 2 :**

Thank you for your valuable comment. As you pointed out, the binding of PD-L1 to PD-1 suppresses T cell function and promotes immune evasion, allowing cancer cells to survive rather than die. The original sentence could lead to a scientific misunderstanding, so we have revised it accordingly.

Please refer to the following for the revised content:

• Introduction: lines 59–64 and 78–82

• System manufacturing and drug delivery methods session: lines 109–110

• Fig. 1(b) (title revised)

Answers : Thank you for your detail reviewer comments for my paper. I revised my manuscript such as follow your comments. Please refer to revise my manuscript. Thank you again.

1. The discussion section should be improved and expanded based on the merits of the study.
2. The STEAM system may be elaborated further for better understanding.
3. Please compare the efficacy of the present system with that in literature.

1. Please ensure that your manuscript meets PLOS ONE's style requirements, including those for file naming. The PLOS ONE style templates can be found at

https://journals.plos.org/plosone/s/file?id=wjVg/PLOSOne_formatting_sample_main_body.pdf

https://journals.plos.org/plosone/s/file?id=ba62/PLOSOne_formatting_sample_title_authors_affiliations.pdf

https://journals.plos.org/plosone/s/file?id=wjVg/PLOSOne_formatting_sample_main_body.pdf and

https://journals.plos.org/plosone/s/file?id=ba62/PLOSOne_formatting_sample_title_authors_affiliations.pdf

2. We note that the grant information you provided in the ‘Funding Information’ and ‘Financial Disclosure’ sections do not match.

When you resubmit, please ensure that you provide the correct grant numbers for the awards you received for your study in the ‘Funding Information’ section.

3. Thank you for stating the following financial disclosure:

This work was supported by the Korea Medical Device Development Fund grant funded by the Korea government (the Ministry of Science and ICT, the Ministry of Trade, Industry and Energy, the Ministry of Health & Welfare, the Ministry of Food and Drug Safety) (Project Number: 1711196789, RS-2023-00252804), and the research work was supported by the GRRC program of Gyeonggi province. [GRRC-Gachon2023(B01), Development of AI-based medical imaging technology].

Please state what role the funders took in the study. If the funders had no role, please state: "The funders had no role in study design, data collection and analysis, decision to publish, or preparation of the manuscript."

If this statement is not correct you must amend it as needed.

Please include this amended Role of Funder statement in your cover letter; we will change the online submission form on your behalf.

4. Thank you for stating the following in the Acknowledgments Section of your manuscript:

This work was supported by the Korea Medical Device Development Fund grant funded by the Korea government (the Ministry of Science and ICT, the Ministry of Trade, Industry and Energy, the Ministry of Health & Welfare, the Ministry of Food and Drug Safety) (Project Number: 1711196789, RS-2023-00252804), and the research work was supported by the GRRC program of Gyeonggi province. [GRRC-Gachon2023(B01), Development of AIbased medical imaging technology].

Animal experiments performed at the HLBI Biostep (Songdo Research Center, Incheon, Republic of Korea), and all procedures are approved by the Institutional Animal Care and Use Committee (BIOSTEP IACUC 23-KE0515).

Seon Min Lee, Kicheol Yoon, and Sangyun Lee contributed equally to this work. Seon Min Lee, Kicheol Yoon, and Sangyun Lee are the co‐first (lead) authors.

Hyun Guy Kang and Kwang Gi Kim are co-corresponding authors.

We note that you have provided funding information that is not currently declared in your Funding Statement. However, funding information should not appear in the Acknowledgments section or other areas of your manuscript. We will only publish funding information present in the Funding Statement section of the online submission form.

Please remove any funding-related text from the manuscript and let us know how you would like to update your Funding Statement. Currently, your Funding Statement reads as follows:

This work was supported by the Korea Medical Device Development Fund grant funded by the Korea government (the Ministry of Science and ICT, the Ministry of Trade, Industry and Energy, the Ministry of Health & Welfare, the Ministry of Food and Drug Safety) (Project Number: 1711196789, RS-2023-00252804), and the research work was supported by the GRRC program of Gyeonggi province. [GRRC-Gachon2023(B01), Development of AI-based medical imaging technology].
Please include your amended statements within your cover letter; we will change the online submission form on your behalf.

5. Your ethics statement should only appear in the Methods section of your manuscript. If your ethics statement is written in any section besides the Methods, please delete it from any other section.

6. When completing the data availability statement of the submission form, you indicated that you will make your data available on acceptance. We strongly recommend all authors decide on a data sharing plan before acceptance, as the process can be lengthy and hold up publication timelines. Please note that, though access restrictions are acceptable now, your entire data will need to be made freely accessible if your manuscript is accepted for publication. This policy applies to all data except where public deposition would breach compliance with the protocol approved by your research ethics board. If you are unable to adhere to our open data policy, please kindly revise your statement to explain your reasoning and we will seek the editor's input on an exemption. Please be assured that, once you have provided your new statement, the assessment of your exemption will not hold up the peer review process.

7. We note that your Data Availability Statement is currently as follows: All relevant data are within the manuscript and its Supporting Information files.
Please confirm at this time whether or not your submission contains all raw data required to replicate the results of your study. Authors must share the “minimal data set” for their submission. PLOS defines the minimal data set to consist of the data required to replicate all study findings reported in the article, as well as related metadata and methods (<https://journals.plos.org/plosone/s/data-availability#loc-minimal-data-set-definition>).

For example, authors should submit the following data:
- The values behind the means, standard deviations and other measures reported;
- The values used to build graphs;
- The points extracted from images for analysis.

Authors do not need to submit their entire data set if only a portion of the data was used in the reported study.
If your submission does not contain these data, please either upload them as Supporting Information files or deposit them to a stable, public repository and provide us with the relevant URLs, DOIs, or accession numbers. For a list of recommended repositories, please see <https://journals.plos.org/plosone/s/recommended-repositories>.
If there are ethical or legal restrictions on sharing a de-identified data set, please explain them in detail (e.g., data contain potentially sensitive information, data are owned by a third-party organization, etc.) and who has imposed them (e.g., an ethics committee). Please also provide contact information for a data access committee, ethics committee, or other institutional body to which data requests may be sent. If data are owned by a third party, please indicate how others may request data access.

8. If the reviewer comments include a recommendation to cite specific previously published works, please review and evaluate these publications to determine whether they are relevant and should be cited. There is no requirement to cite these works unless the editor has indicated otherwise.
